# How can we accelerate progress on civil registration and vital statistics?

**DOI:** 10.2471/BLT.18.211086

**Published:** 2018-04-01

**Authors:** Carla AbouZahr, Martin W Bratschi, Daniel Cobos Muñoz, Romain Santon, Nicola Richards, Ian Riley, Philp Setel

**Affiliations:** a Bloomberg Data for Health Initiative, 6 Chemin des Fins, 1218 Geneva, Switzerland.; b Vital Strategies, New York, United States of America.; c Health Systems and Policy Group, Swiss Tropical and Public Health Institute, Basel, Switzerland.; d Melbourne School of Population and Global Health, University of Melbourne, Melbourne, Australia.; Correspondence to Carla AbouZahr (email: Abouzahr.carla@gmail.com).

The *Bulletin of the World Health Organization* will publish a series of papers drawing attention to the contribution the health sector can make to strengthening civil registration and vital statistics systems and increasing birth and death registration coverage. Key information recorded by the health sector on the occurrence and circumstances of births and deaths, including causes of death, should be harnessed by national civil registration agencies to increase notification and official registration. This would improve the timeliness, completeness and accuracy of vital statistics.^1^ To enable this outcome, stakeholders in civil registration, health, registration, statistics and identification management must collaborate and work together to re-engineer the birth and death registration processes and build information and data sharing into civil registration and vital statistics systems.

Two sustainable development goal (SDG)^2^ targets and related indicators refer to improving civil registration and vital statistics: target 16.9, provide legal identity for all, including birth registration, and target 17.19, the indicator for which is the proportion of countries that have achieved 100 percent birth registration, and 80 percent death registration. 

To reach universal civil registration of births, deaths, marriages and other vital events for all people by 2030, the World Bank and the World Health Organization (WHO) have developed an investment plan for scaling up global civil registration and vital statistics systems.^3^ This ambitious plan acknowledges the multiple roles of such systems. First, the registration of vital events provides people with documented evidence of identity, civil status and family relationships, thereby enabling them to participate in economic, social and political processes, make claims of citizenship and access social protection and benefits. Second, the information collected by the civil registration and vital statistics system enables the continuous updating of population registers, associated registers such as electoral rolls, and well-designed identification systems that are central to national administration and security. Third, the registration of births and deaths generates a wealth of population data that underpin policy and planning across many sectors.

Although progress has been made, increased political will, capacities and resources are needed to achieve universal registration. In 2013, 45% of global deaths were registered compared with 28% in 1970, an increase of 60% over 43 years.^4^ However, only 40 of 194 countries reported high quality cause-of-death data to WHO and two out of three people live in countries where cause of death data quality is inadequate for public health decision-making or to monitor the SDG health targets. In 2015, the births of nearly one-quarter of all children younger than five years had never been registered.^5^ To attain the SDG targets that are related to civil registration and vital statistics, under-five birth registration will need to increase by 33% and death registration will need to increase by 77%, an annual average increase of over 5%. Monitoring many of the SDGs will depend on the availability of continuous, detailed, and local-area-specific statistics that only civil registration systems can provide.^6^

In the upcoming series of perspectives in the *Bulletin of the World Health Organization*, countries, health programmes and agencies will offer some potentially transformative suggestions to capture synergies across sectors; these proposals will follow three strategic approaches. First, systems transformation brings together multiple stakeholders to review and revise legal, regulatory, policy, administrative and statistical processes.^7^ Health workers are aware of the occurrence of births and deaths and routinely collect relevant information and data as part of the provision of care. Health officials should be delegated to notify these vital events to the civil registrar, capturing and transmitting information using agreed protocols in a systematic and standardized way. Second, technological innovation is needed to improve the speed and reliability of vital events registration and streamline data flows, while simultaneously enhancing the experience of individuals and families.^8^ Rationalizing and streamlining health information flows will help reduce duplication, improve quality and enable sharing with other government agencies. Third, people are the core beneficiaries of civil registration and vital statistics. This implies alleviating the burden on families of reporting vital events through delegation of responsibilities to health officials. People centeredness also involves active outreach to disadvantaged and hard-to-reach populations, comprising everyone in a defined territory, including non-nationals. The health sector, through its widespread network of trusted community agents and its mission of service provision, is well-positioned to support such outreach. The health sector also has much to gain from closer collaboration with such systems, because they generate data that are needed for local health planning and service delivery. In addition, feedback to health from civil registration agencies can help identify vital events that take place outside the health system and areas that need improved health and social services.
